# How Minority Parents Could Help Children Develop Healthy Eating Behaviors: Parent and Child Perspectives

**DOI:** 10.3390/nu12123879

**Published:** 2020-12-18

**Authors:** Chishinga Callender, Denisse Velazquez, Meheret Adera, Jayna M. Dave, Norma Olvera, Tzu-An Chen, Shana Alford, Debbe Thompson

**Affiliations:** 1 USDA/ARS Children’s Nutrition Research Center, Department of Pediatrics, Baylor College of Medicine, 1100 Bates Street, Houston, TX 77030, USA; Chishinga.Callender@bcm.edu (C.C.); himedenisse@gmail.com (D.V.); msa8@rice.edu (M.A.); jmdave@bcm.edu (J.M.D.); 2 Psychological, Health, and Learning Sciences Department, University of Houston, 3657 Cullen Boulevard Room 491, Houston, TX 77204, USA; nolvera@central.uh.edu; 3 HEALTH Research Institute, University of Houston, 4849 Calhoun Road, Houston, TX 77204, USA; ann0516@gmail.com; 4 Common Threads, 222 W. Merchandise Mart Plaza, Suite 1212, Chicago, IL 60654, USA; salford@commonthreads.org

**Keywords:** minority, parents, children, obesity, prevention, diet, nutrition promotion, Black/African American, Hispanic, qualitative

## Abstract

Minority children and children living in under-resourced households are at the greatest risk for obesity and diet-related disparities. Identifying effective strategies to reduce these risks is an important step in child obesity prevention. Parents influence the home environment and play a critical role in child obesity prevention. Eighteen parent–child dyads living in under-resourced Houston area communities participated in a mixed methods study (online surveys, telephone interviews). The purpose of the research reported here was to conduct a secondary analysis of the qualitative data to explore Black/African American and Hispanic parent and child perspectives of the ways in which parents could help their children make healthy food choices. Descriptive statistics were calculated for parent and child demographic characteristics; hybrid thematic analysis was used to code and analyze the interview transcripts. Frequencies were calculated for children’s interview responses to rating scales and the grade they gave their eating habits. Mothers’ responses were grouped into two broad categories: facilitators (modeling, availability, and teaching) as ways parents could help their child eat healthy, and barriers (lack of time, cost of healthy foods, and lack of knowledge) to helping their child eat healthy. Alternatively, child responses focused on ways in which parents could provide support: environmental support (home availability, home cooking, and introducing new foods) and personal support (providing child choice, teaching, and encouragement). Most children reported that eating healthy was easy, and most rated their personal eating habits as an A or B. These findings suggest that understanding the perspectives of Black/African American and Hispanic parent–child dyads can provide insight into the development of culturally and economically relevant healthy eating strategies and interventions for families living in under-resourced communities.

## 1. Introduction

The high prevalence of child obesity in the United States is a significant public health issue [1], with the highest prevalence among Black/African American and Hispanic children [2,3] and children living in low-income households [4]. Identifying effective strategies to reduce the risk of child obesity and related disparities is an important step in child obesity prevention [5].

Several factors influence child obesity risk, including diet, physical activity, and sedentary behavior [6,7]. Through excessive caloric intake, diet plays a significant role in obesity risk [6,7]. Several factors influence dietary intake, including the home food environment [8], parenting styles [9], family meals [9], personal food preferences [10], and household income [11]. Furthermore, the diet quality of children in the United States falls below the national dietary recommendations [12].

Similar to obesity-related disparities, diet-related disparities exist, and it is imperative to address them as well. Racial and ethnic minorities (i.e., Black/African American, Hispanic) experience diet-related disparities and tend to have poorer diet quality compared to their white counterparts [13]. In addition, these disparities exist in low-income populations, as poor diet quality is associated with socioeconomic status [14]. For example, a study found that lower-income individuals ate lower-quality foods than their higher-income counterparts [14]. Families living in under-resourced communities are more likely to be exposed to advertising of less healthy foods and beverages [15], have more access to fast food restaurants [16], and less access to stores with affordable healthy foods [17]. Thus, minority, under-resourced families may be at the greatest risk for experiencing diet-related disparities. Understanding how these factors influence child dietary behaviors is essential in developing effective interventions to reduce poor diet quality for children.

Parents exert a strong influence on the home food environment [9,18]. Therefore, they play an important role in child obesity prevention. It is critical for parents to encourage children to engage in healthy dietary behaviors at an early age to reduce children’s risk of obesity and related diseases and to help them develop and maintain a healthy lifestyle into adulthood. Previous research identifies the challenges that families living in under-resourced communities experience accessing healthy foods [11,19]. In addition, previous studies have identified parents’ perspectives on strategies to promote healthy eating behaviors [20,21,22,23]. However, few studies have explored perspectives of racial and ethnic minority parent–child dyads regarding ways parents can help their children practice healthy eating behaviors. The perspectives of parents and children in minority and under-resourced communities are essential for developing effective, culturally appropriate, and acceptable obesity prevention and nutrition promotion interventions. Thus, the purpose of this research was to expand the voices of minority families in the literature by investigating both parent and child perspectives of the ways in which parents can help their children make healthy food choices.

## 2. Materials and Methods

This is a secondary data analysis of data from a larger, mixed methods study designed to identify thoughts, expectations, and preferences of parents and children toward cooking and nutrition education programs promoting healthy eating. This paper was guided by the following research question: From the perspectives of both parents and children, how can parents help their children make healthy food choices?

### 2.1. Design

This study re-examined qualitative data from a larger mixed-methods study. The protocol was approved by the institutional review board at Baylor College of Medicine (H-44683).

### 2.2. Study Participants

Inclusionary criteria for the families included: child 8–13 years old; parent/guardian of a child 8–13 years old; both parent and child fluent in English or Spanish; child receives and/or is eligible for free or reduced price meals at school; healthy (i.e., no physical, health, or medical condition that would affect diet or participation in a telephone interview or focus group); parent and child living in the same household; the parent has primary responsibility for family food shopping/acquisition and/or meals; transportation to focus group (focus group participation only); families also needed to be willing to provide contact information, to participate in study activities, and have focus group/telephone interview audio recorded. Exclusionary criteria included unwillingness to have the telephone interview or focus group recorded and to take photos for the study.

### 2.3. Recruitment

Families living in under-resourced communities in the Greater Houston, TX metropolitan area were recruited for this study. A more detailed description of the recruitment procedures is reported elsewhere [24]. Briefly, recruitment started in early May 2019 and ended in mid-August 2019. Recruitment methods included contacting potential families from the volunteer database at the USDA/ARS Children’s Nutrition Research Center (CNRC) and referrals from recruited families. All families provided written informed consent prior to participation. During the consent process, parents had the option for their family to participate in either separate telephone interviews or focus groups in their preferred language (English or Spanish). All families chose to participate in telephone interviews.

### 2.4. Data Collection

Data collection for the larger study began in June 2019 and ended in October 2019. In that study, parents and children completed online surveys, took photographs of situations that made it easy or hard to make healthy food choices, and completed telephone interviews. Surveys were available in English and Spanish, and telephone interviews were conducted in participants’ preferred language (English or Spanish). Trained research coordinators conducted semi-structured telephone interviews [25]. Interviews were scripted and contained open-ended, non-leading questions; probes and prompts were used to clarify, expand, and understand responses. Separate scripts guided parent and child interviews. This paper reports demographic data, as well as parent and child responses to the following interview questions: “How can parents help their children to eat healthy?” (parents); “How could your parents help you make healthy food choices at home?” (child). To explore children’s perspectives related to their personal eating habits, child responses to the following interview questions are also reported: (1) “On a scale of 1 (hard), 2 (not hard or easy), 3 (easy), how easy or hard is it to eat healthy?” (2) “What grade would you give your eating habits (i.e., A, B, C, D, F like in school grades)?” Each interview was digitally recorded, transcribed, and reviewed for accuracy prior to analysis. 

### 2.5. Data Analysis

#### 2.5.1. Surveys

A single dataset was created by combining the English and Spanish language surveys. Parent and child data were analyzed separately. Descriptive statistics (frequencies, percentages) were calculated for demographic and household characteristics.

#### 2.5.2. Interviews

Two independent coders used hybrid thematic analysis to code and analyze verbatim transcripts [26]. Transcripts were reviewed for accuracy prior to analysis. To provide structure to the coding process, transcripts were initially coded using a priori codes guided by the research question. Emergent codes were generated as transcripts that were reviewed and coded to provide flexibility to the coding process and ensure that parent and child perspectives were fully captured. After coding was complete, the codes were reviewed for relevance to the research question. During this process, some codes were dropped, and others were grouped into categories based on their similarities. Then, categories were reviewed and grouped into higher-order categories as appropriate. Analysis was conducted on English language transcripts (i.e., English language transcripts and translated Spanish language transcripts). Separate parent and child codebooks were maintained and routinely updated to reflect new emergent codes, definitions, and key decisions. Frequencies were calculated for children’s responses to rating scales and the grade they gave their eating habits. Their reasons for selecting a rating or grade were used to expand the findings and provide additional insight.

Verbatim quotes, used to support qualitative findings, were labeled as follows: P = Parent; C = child; A = Black/African American; H = Hispanic. To help differentiate the quotes, a number (from 1–18) was assigned to each parent/child dyad.

## 3. Results

### 3.1. Family Characteristics

Eighteen parent–child dyads enrolled in the study. All participating parents were mothers (100%), mostly 40–49 years old (61%), Black/African American (56%), Hispanic (44%), and married/living with significant other (61%). Over half of the children were female (56%) and Black/African American (56%). Half of the children’s ethnicity (50%) was reported as Hispanic by the mothers. The ages of children participating included 8–10 years old (22%) and 11–13 years old (78%).

The highest level of household education varied, with 33% of families having less than a college education, 33% having some college coursework, 22% having a college degree, and 11% having a postgraduate degree. Annual household income for the majority of families (66%) was below $41,000. To add context, 39% of mothers reported two adults in the household, and 50% reported having two children under the age of 18 living in the household. Although the majority of mothers reported high/marginal food security (89%), more than half reported using one to three food assistance programs (67%). The majority of families (61%) spoke mostly English at home. All families recruited for the study met the inclusionary criteria. 

### 3.2. Interview Findings

**Figure 1:**
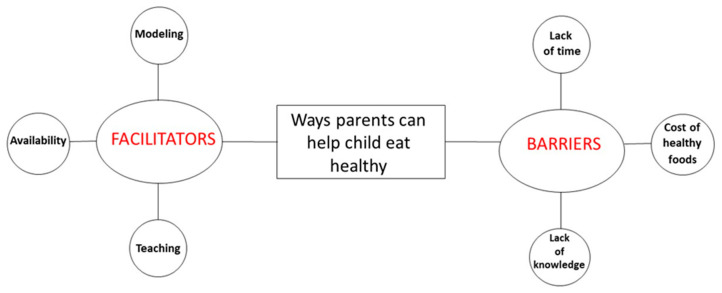
Visual representation of mothers’ perspectives on ways parents can help their children eat healthy. (Note: Red font indicates the categories. Bold black font indicates the supporting categories that emerged.)

**Figure 2:**
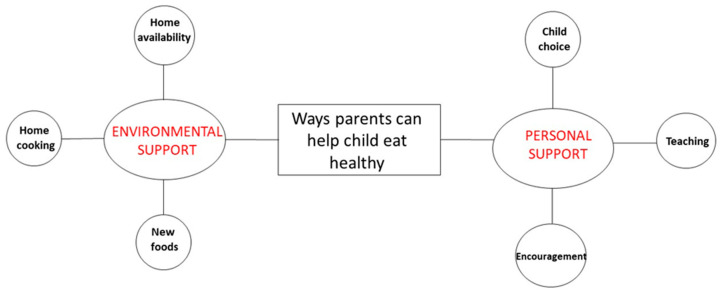
Visual representation of children’s perspectives on ways parents can help their children eat healthy. (Note: Red font indicates the categories. Bold black font indicates the supporting categories that emerged.)

Interviews ranged from an average of 43 min (children) to 56 min (mothers). As previously stated, the a priori codes were generated from the research question prior to coding. Then, emergent codes were added throughout coding based on parents and children responses. After coding all transcripts, the emergent codes were reviewed, collapsed, and grouped into categories. Parents’ responses were grouped into two categories: facilitators (what made it easy to help their child make healthy food choices) and barriers (what made it difficult to help their child make healthy food choices). Children’s responses were also grouped into two categories: environmental support (how parents create healthy eating choices at home) and personal support (how parents actively influence the child to eat healthy). The categories are presented in Figure 1 (parent) and Figure 2 (child).

#### 3.2.1. Parents

When parents were asked, “How can parents help their child eat healthy?” two main categories emerged: facilitators and barriers to helping their child eat healthy (Figure 1). Each is described in more detail below in order of prevalence and presented in Table 1.

**Facilitators.** Three ways parents could help their children make healthy food choices were mentioned: (1) modeling healthy eating behaviors, (2) having healthy foods easily available for the child (availability), and (3) teaching the child about healthy eating. Several mothers shared that modeling healthy eating is beneficial to a child: *“By eating healthy themselves. Kids follow what we do. And if they see a parent eating healthy, they are more likely to follow in their parent’s footsteps”* (P.15A). Mothers shared examples of ways to make healthy foods easily available for their children. One mother mentioned, *“I think that… the healthy way depends on what kind of food. Because I try to use those tricks in the soup so that they eat it. So that they can eat healthier. And with fruit, I try to keep as many containers full of fruit as possible, so that they don’t have to wash them, so that they can see it [as] appetizing there, in the refrigerator”* (P.6H). A few mothers shared their perspective about teaching healthy eating habits to their children. For example, one mother taught her children about the benefits of healthy eating: *“By explaining to them that our food is our medicine, and our medicine has to be our food, because we are what we eat. By showing them the effects that diseases caused by unhealthy food have on different people”*(P.17H). Another mother shared teaching children about preparing healthy foods: *“I think that as a parent, we help them package up food portions, be it in Ziploc bags or in Tupperware, teaching them how to divide up food and have it prepared for when they come back from school, or some sport, grab it and eat”* (P.18H).

**Barriers.** Parents mentioned three types of barriers to helping children eat healthy: (1) lack of time, (2) cost of healthy foods, and (3) parent’s lack of knowledge about healthy eating and cooking. Lack of time to prepare or serve healthy foods was noted as a barrier by several mothers. For example, one mother shared how time affects working mothers: *“Sometimes, you know, when the mother works or both parents work, especially the mother, there is no time to prepare, like, maybe healthy food. So, maybe, you know, ‘cause you need to cook or to grill or whatever. So the things that are not healthy in a can or fast food that you buy, you know, it’s easier, so the family ends up eating unhealthy because their mother would be tired and all that. But I think time really plays a role, especially if the mother is working”* (P.2A). However, stay-at-home mothers did not see lack of time as a barrier: *“Time? No, not right now because the children are on vacation. I’m here with them, I don’t work. So, of course I can go to the store to buy the ingredients or whatever I need. No, time is no obstacle”* (P.12H).

The high cost of healthy foods was also seen as a barrier for some mothers: *“Economics. If a parent feels as though they don’t have the money for the healthy food, they’re likely to buy what’s cheap”* (P.15A). One mother shared her personal experience with household finances impacting eating choices for her family: *“Yes, one of the first obstacles is that sometimes... the day you don’t eat healthy at home is for lack of... it’s because our budget is really low. But, we try to do the best we can”* (P.3H).

Knowledge also emerged as a barrier to helping children make healthy food choices. For example, one mother shared how lack of knowledge of “…how to cook [or] prepare… food” was a barrier to helping children eat healthy (P.11A). Another shared how some parents are not knowledgeable about healthy ingredient substitutions for meals they enjoy cooking: *“And they may not know that you can cook the foods you love but you don’t have to use—you can use different ingredients to get the flavor and the taste you want”* (P.1A).

#### 3.2.2. Children

When children were asked, “How could your parents help you make healthy food choices at home?” two main categories emerged: environmental and personal support by parents (Figure 2). Each is described in more detail below in order of prevalence and presented in Table 2.

**Environmental support.** Three ways parents could encourage a child’s healthy eating choices in the home environment were mentioned by children: (1) home availability (i.e., healthy foods easily available at home), (2) home cooking, and (3) introducing new foods. Several children suggested that their parents could make healthy foods more available. For example, one child shared, *“Maybe by like buying more healthier food like more fruits and vegetables”* (C.15A). Another child shared how their mother makes healthy foods available at home: *“Well, she would help me by like at home, we would find things in the house that would be healthy. Like, fruit in yogurt and stuff. Sometimes, we’ll make, like, smoothies, like, but they would have, like, lots of vegetables. Well, like, vegetables and things inside. Plus, we have a lot of vegetable things we cook with”* (C.16A).

For home cooking, children also noted that preparing foods in a healthy way would be helpful: *“Or maybe when we cook not putting as much grease and butter and unhealthy foods like that”* (C.15A). Some children also suggested including more vegetables in meals. For example, one child shared, *“…my mom and my dad could help me in the kitchen with different types of vegetables and different types of protein, things that I need”* (C.8A).

Introduction to new or different foods was also suggested by children: *“And—but I think to help us more she could, like, start influencing us by giving us more creative things and different foods that sometimes we’ve never tried. Because if you keep eating the same thing over and over, even though it’s healthy, it might get boring and you don’t want to eat it anymore”* (C.10H). Children shared examples of how their parents introduced new foods, including through sharing new recipes, watching their mothers cook, and cooking foods from different cultures. For example, one child shared her experience with cultural foods: *“Mainly, my mom influences me to try new food because since she’s, like, from… she’s from Mexico, she, like, influenced me to try a lot of new foods from other places”* (C.10H). It is worth noting that a majority of children identified their mothers as an influence to try new foods.

**Personal support.** Children mentioned three types of personal support parents could use to help children eat healthy: (1) giving the child choice, (2) teaching, and (3) providing encouragement. Giving the child choice (i.e., opportunities to choose foods, preparation methods, and meals) was recommended by a few children. For example, one child shared how parents could ask questions about making healthy food choices: *“I think if they asked more about what type of food we want to eat that is healthy”* (C.2H). A few children expressed interest in making choices at the grocery store: *“Going with her to the grocery store to pick out the vegetables and fruits I want”* (C.7H).

For teaching, a child was interested in her mother teaching her to cook healthy: *“My mom could teach me how to cook healthier meals so that way when I [get] hungry I could just cook that and I would be able to eat healthy”* (C.13A). A few children shared that their mothers taught them about cooking through showing them: *“Mom teaches us how she does things. And then my mom does it”* (C.12H). For encouragement, one child shared how his mother encouraged him to make healthy snack choices: *“Well, sometimes… because most of the time, I like to walk to the store and buy chips, but my mom told me that I shouldn’t be walking to the store and getting chips because chips are unhealthy, so she just doesn’t give me the money. She says if you go to the store you better come back with some fruit cups or something.”* A child also shared how her mother encouraged her to make healthy choices at restaurants: *“So when we go places, like, for example Chick-fil-A and all that stuff that has a lot of grease, she encourages me to try, like, salads and stuff”* (C.16A). Simple words of encouragement with reasoning were also noted as helping a child with eating healthy: *“She says you won’t know if you won’t taste it”* (C.11A).

#### 3.2.3. Children’s Perspectives on Making Healthy Dietary Choices 

##### Ease and Difficulty of Healthy Eating

When children were asked, “on a scale of 1 (hard), 2 (not hard or easy), 3 (easy), how easy or hard is it to eat healthy,” the following ratings were reported: rating of 1 (*n* = 3), rating of 2 (*n* = 5), and rating of 3 (*n* = 10). Reasons for choosing the ratings varied. For example, one child who gave a rating of 1 noted eating habits as a reason healthy eating was difficult for the general population: *“Well, I think it’s hard to eat healthy because some people might be so used to eating unhealthy that they’ll just like they’re are going to keep eating unhealthy. But they might eat something healthy not very often, but they’ll probably eat it every once in a while”* (C.1A). Children shared a variety of reasons for choosing a rating of 2. A child mentioned how temptation can influence eating healthy: *“Because it’s not hard to eat healthy, but it’s not also easy to eat healthy. Because if it’s easy to eat healthy but if you see stuff that’s unhealthy and it looks good or smells good, you might want it”* (C.9A). Another shared how types of foods can influence eating choices: *“I chose two because it can be easy sometimes but not always is, but it’s not hard either. It depends on the food I’m eating and the taste”* (C.14H).

Children also shared a variety of reasons for choosing a rating of 3. A few children noted parental and/or family influences on their eating choices. For example, one child shared how the entire family encourages healthy eating: *“Because in my household, my family, like my sisters, especially my older sister, my dad, my mom, they really, like, encourage us to eat foods that don’t contain things that are bad for your body and healthy things”* (C.10H). Another child shared that her mother *“buys fruits and vegetables”* (C.4A). Some children also noted their eating habits as a reason for choosing a rating of 3: *“Because I like eating lots of apples and salads so it’s much easier to do that”* (C.11A) and *“Because I normally eat healthy”* (C.13A). The benefits of healthy eating were also mentioned as a reason: *“Three cause it doesn’t really matter really what you eat, you have to have a lot of—I mean, not a lot, less calories and you have to have a lot of vegetables and stuff like lettuce, carrots, tomatoes, a lot of things that will help your body”* (C.16A).

##### Personal Eating Habits Assessment

When children were asked, “What grade would you give your eating habits?” they reported the following grades: A (*n* = 3), B (*n* = 10), C (*n* = 2), and D (*n* = 2). Reasons varied for choosing the grades. For example, a child who reported an A for their eating habits mentioned his mother’s cooking: *“‘Cause most of the things I eat, my mom cooks and she cooks a lot with a lot of vegetables and stuff so to help be healthy”* (C.16A). Similarly, a child with an “A” grade mentioned eating healthy foods at home and school: *“At school, the food is very healthy. They give us balanced meals, but sometimes the taste just isn’t so great. Sometimes it’s a bit hard to eat. And at home it’s an A, because most of the time, almost all the time it’s very healthy and the taste is good so it’s easier to eat”* (C.14H). Another described their favorite types of foods, while also noting fruit purchases made by his mother as the reason for reporting an A.

Several children who reported a “B” or “C” grade for their eating habits mentioned mixed eating habits (i.e., eating healthy and unhealthy foods) as a reason. One child shared the following: *“Because I don’t, like, all the time it won’t always be healthy food but sometimes it’s healthy food. And then sometimes I do have sweets now and then*” (C.8A). One child noted differences in foods eaten in the home and school environment as a reason for a “B” grade: *“Because I eat great at home, but at school not that well”* (C.17H). Interestingly, one child compared their eating habits to their friends as the reason for a “B” grade: *“…because I’m not that healthy that I over overpass everything. But I do consider myself eating healthier than a lot of my friends”* (C.10H). Both children who reported a “D” grade shared a lack of vegetable consumption as the reason.

## 4. Discussion

The purpose of this research was to investigate the perspectives of minority parents and children on the ways in which parents could help their children make healthy eating choices. Although a few studies [27,28] have presented interview findings from parent–child dyads regarding healthy eating, this study is unique in that it highlights both parent and child perspectives of the ways parents could help their children eat healthy. This study is also distinctive as it presents interview findings from children about the ease and difficulty of eating healthy and an assessment of their personal eating habits.

For mothers in this sample, modeling, availability, and teaching served as the main facilitators for parents to help children practice healthy eating behaviors. Similar to our research, parental roles of modeling healthy eating behaviors [29,30], making healthy foods available [8,29], and teaching about healthy eating [29] have been reported by parents in other studies. Previous studies have also found that availability of healthy foods [8,31] in the home and modeling [31] have been associated with child’s diet quality. Contrary to previous research, child’s weight was not mentioned as a motivation to encourage healthy eating behaviors in children [27]. These findings suggest that interventions promoting healthy eating behaviors for minority children living in under-resourced communities should promote strategies such as modeling, making healthy foods available at home, and teaching children how to eat and cook healthy, while avoiding an emphasis on child’s body weight.

For mothers in this sample, lack of time, cost of healthy foods, and lack of knowledge were highlighted as barriers to helping children practice healthy eating behaviors. Lack of time to prepare healthy meals [27,30,32,33,34] is consistently reported by parents as a barrier to healthy eating. This could be due to parents from under-resourced families working long hours and/or having multiple jobs [32]. However, a few mothers in our study did not report a lack of time as a barrier due to their role as stay-at-home mothers. Similar to mothers in our study, parents from previous studies, including those with limited income, also identified the cost of healthy foods as a barrier and also believed that healthy eating is expensive [21,27,30,32,33,34]. Lack of knowledge was also reported as a barrier by parents with limited income [32] and minority families [27]. These findings highlight the need for developing parent-focused interventions for minority and under-resourced communities with a focus on teaching time management strategies for preparing healthy meals, providing affordable options for purchasing healthy foods (e.g., purchasing fresh fruits and vegetables in season), and promoting nutrition and cooking skills knowledge.

Children reported that home availability, home cooking, and new foods were environmental support factors to be practiced by parents to help children eat healthy. Home availability [35], home cooking [36], and willingness to try new foods [37] were reported by children in other studies as ways in which parents helped them make healthy eating choices. In our study, both mothers and children reported that home availability of healthy foods can support the creation of a healthy home food environment. The availability of healthy foods is consistently supported in the literature as an agent for changing children’s dietary behaviors [9,29]. In addition to availability, it is imperative that nutrition education/promotion interventions are inclusive of strategies on home cooking (e.g., healthful preparation methods, cooking with the child) and how to introduce new foods.

Children also reported that giving the child choice, teaching, and encouragement were personal support factors to helping children eat healthy. These factors were found to be similar to concepts in previous research, including instrumental and emotional social support for healthful eating from the parent’s perspective [38] and positive parental parenting (i.e., feeding practices) [39]. Comparable to our study, middle school aged children in the northeastern United States noted that their food choices were influenced by their parent’s guidance [40]. Interestingly, child choice was seen differently by children in Spain, as they did not have a choice in how the foods were prepared, but they were given an option for the amount and type of foods eaten [37]. Similar to our study, children noted that parents shared advice on fruit and vegetable consumption and encouraged them to eat foods prepared at home instead of foods prepared outside of the home [36].

Furthermore, similar to our study, teaching and encouragement were identified as active guidance in five research studies, and a positive relationship was found with vegetable consumption in one study [29]. In our study, the environmental and personal support factors were primarily influenced by mothers. The literature consistently supports mothers influencing the eating behaviors of their children [9]. It is important to note that both children and mothers mentioned teaching as a way parents can help their children eat healthy. Future research should explore the role of fathers in the environmental and personal support factors for children’s dietary behaviors. Based on the above findings, intervention programs should include strategies for parents on ways to offer choices in healthy foods, teaching about them (e.g., types of healthy foods, benefits of healthy eating), and encouraging their child to eat them.

Our study highlighted another contribution to the literature: children’s perspectives on the ease and difficulty of eating healthy. A variety of reasons (i.e., eating habits, benefits of healthy eating, and temptations) were mentioned in considering healthy eating choices. It is critical to note that a few of the children’s responses reflected parental influences (i.e., maternal encouragement and purchasing of fruits and vegetables). Children’s perspectives on their personal eating habits also contribute to the literature. A majority of the children rated their eating habits highly, while only a few rated their eating habits below a B. Similar to reasons for the ease and difficulty of eating healthy, children noted parental influences (i.e., home cooking, mother purchasing fruits) attributing to their eating choices. Comparable to our study, children in England shared parental influences on their eating choices [41]. Food choices at school, mixed eating habits, and a lack of vegetable consumption were also mentioned as reasons for the personal eating habits grade. Further investigation is needed into children’s perspectives on their personal eating habits. Although parents believed that they needed to make ample effort to help their children eat healthy, children simply wanted environmental modifications (e.g., having healthy foods available, home cooked meals, introduction of new foods) and personal support from parents (e.g., the freedom to make their own food choices, lessons on healthy eating, healthy eating encouragement). This suggests that future interventions to encourage healthy eating among minority children and children living in under-resourced communities should focus on encouraging these types of changes.

The strengths of this study included semi-structured interviews and interviewers trained in qualitative methods. One limitation included the differences in mothers’ responses to the interview question; there was a lack of clarity in whether mothers were speaking of their personal experiences or their beliefs about the experiences of other parents. A second limitation included children’s responses to the interview questions on the ease and difficulty of healthy eating; the data did not lend itself to hybrid thematic analysis. Despite the limitations, the findings provide an opportunity to identify parent and child perspectives of the ways in which parents can help their children make healthy dietary choices.

## 5. Conclusions

Insights from minority parents and children are critical to the development of effective child obesity prevention and nutrition education programs for families living in under-resourced communities.

Strategies centered on environmental and personal support are needed for parents to help their children develop and practice healthy eating behaviors. It is important that child nutrition promotion strategies tailor to the perspectives of families with the priority of reducing diet-related disparities among racial/ethnic minorities and under-resourced communities.

## Figures and Tables

**Table 1:** Categories and subcategories from qualitative data analysis of interviews with parents on ways parents can help their children eat healthy.

Categories	Subcategories	Definitions
Facilitators	Modeling Availability Teaching	Modeling healthy eating behaviors Having healthy foods easily available Teaching healthy eating behaviors
Barriers	Lack of time Cost of healthy foods Lack of knowledge	Lack of time to prepare or serve healthy foods High cost of healthy foods Lack of knowledge about healthy eating and cooking

**Table 2:** Categories and subcategories from coding of interviews with children on ways parents can help their children eat healthy.

Categories	Subcategories	Definitions
Environmental Support	Home availability Home cooking New foods	Healthy foods easily available at home Cooking foods at home Introduction to new or different foods
Personal Support	Child choice Teaching Encouragement	Giving child the opportunity to choose foods, preparation methods, and meals Teaching healthy eating behaviors Encouraging child to practice healthy eating behaviors
